# Chronic Administration of Marinobufagenin in Mice Causes Hyperlocomotion and Decrease in Anxiety by Altering Monoamine Turnover Unaccompanied by Motor Deficits or Oxidative Stress

**DOI:** 10.3390/ijms27135713

**Published:** 2026-06-24

**Authors:** Rogneda B. Kazanskaya, Arina O. Lobaskova, Anna D. Iushina, Denis A. Abaimov, Olga I. Kulikova, Anna B. Volnova, Vassiliy Tsytsarev, Alexander V. Lopachev

**Affiliations:** 1 Institute of Translational Biomedicine, St. Petersburg State University, Universitetskaya Nab. 7/9, 199034 St. Petersburg, Russia; st107445@student.spbu.ru (A.O.L.); usinaanna2000@gmail.com (A.D.I.); a.volnova@spbu.ru (A.B.V.); 2 Biological Department, St. Petersburg State University, 199034 St. Petersburg, Russia; 3 Russian Center of Neurology and Neurosciences, 125367 Moscow, Russia; abaidenis@yandex.ru (D.A.A.); posibilidad@mail.ru (O.I.K.); 4 Johns Hopkins University, Baltimore, MD 21218, USA; tsytsarev@jhu.edu

**Keywords:** marinobufagenin, Na^+^,K^+^-ATPase, dopamine, norepinephrine, serotonin, oxidative stress, mania, bipolar disorder, gait

## Abstract

Cardiotonic steroids (CTS) can modulate central nervous system function through their interaction with the Na^+^,K^+^-ATPase, affecting dopaminergic transmission. While the CTS ouabain is known to induce mania-like behavior and oxidative damage, the effects of other CTS are less clear. This study examined the effects of 14-day intracerebroventricular administration of 1.5 μL 100 μM marinobufagenin (MBG) on locomotion, gait, monoamine metabolism, and oxidative stress markers (MDA, SOD, catalase, MAO-B) in C57BL/6 mice. Chronic MBG caused increased locomotor activity and time spent in the center of the open field. Unlike ouabain, chronic MBG did not impair motor function, evaluated via gait analysis. MBG elevated striatal MAO-B activity and reduced prefrontal MDA levels, with no changes in SOD or catalase, indicating that it did not cause oxidative stress. However, it did affect dopamine and serotonin metabolism. Monoamine tissue content evaluation on day 15 showed increased dopamine turnover in the striatum and brain stem, and a decrease in the thalamus. Norepinephrine levels increased in the striatum and hippocampus. Serotonin turnover increased in the prefrontal cortex. These results indicate that chronic MBG increases locomotion and reduces anxiety-like behavior through region-specific modulation of dopaminergic and serotonergic signaling distinct from that caused by ouabain.

## 1. Introduction

Cardiotonic steroids (CTS) are a class of compounds with a conserved steroid core and a 17-position lactone ring—either a five-membered cardenolide or a six-membered bufadienolide—often glycosylated at the C3 position [1]. Long before the molecular mechanisms behind their action were elucidated, CTS such as digoxin were mainstay treatments for heart failure; even earlier, organisms known to synthesize these compounds, such as foxglove and various toads, were employed in traditional medicine [1]. Systematic insight into their action began in 1957 with Jens Skou’s discovery of the Na^+^,K^+^-ATPase [2], which was subsequently identified as the specific inhibitory target for CTS [3]. Until the 1980s, these compounds were viewed strictly as exogenous and research was conducted under the paradigm that CTS serve solely as tools to inhibit Na^+^,K^+^-ATPase activity.

This perspective underwent a fundamental shift with the discovery of endogenous CTS within mammals. The history of their identification across various organs, tissues, and biological fluids has since been extensively reviewed [4,5,6,7]. It was later found that CTS can not only inhibit the Na^+^,K^+^-ATPase, but also activate intracellular signalling cascades when binding it [8]. Since then, a multitude of physiological and pathophysiological processes involving CTS have been described in mammals, along with their underlying mechanisms of action [6,8].

While much of the existing literature characterizes the role of CTS across various organs, their function within the central nervous system (CNS) remains less defined. In the CNS, the primary target of CTS—the Na^+^,K^+^-ATPase—exhibits its highest degree of molecular diversity, represented by the α1, α2, and α3 isoforms. Each isoform possesses a distinct physiological role and precise cellular localization [9]. In the adult mammalian brain, α2 is primarily expressed in glial cells alongside the ubiquitous α1 [9]. Neuronal expression, however, involves α1 and α3 in ratios that vary according to a neuron’s firing frequency. In fast-spiking interneurons, such as parvalbumin-positive cells, α3 is the predominant isoform, whereas less active neurons rely primarily on α1 [9]. This divergence is driven by the specific kinetic profile of α3, which features a higher turnover rate but lower affinity for Na^+^—a specialized adaptation necessary for the rapid restoration of ionic gradients following high-frequency action potential bursts [10].

Although investigations into the effects of ouabain on the CNS—specifically regarding dopamine (DA) release and reuptake—emerged alongside studies in other tissues [11,12,13], a more granular understanding of CTS function in the brain was catalyzed by two key developments: clinical reports of psychiatric disturbances in patients following high-dose digoxin treatment [14] and the isolation of an endogenous CTS from the bovine hypothalamus [15]. These findings led to the observation that intracerebroventricular (ICV) administration of ouabain in rats induces a mania-like behavioral phenotype [16]. Since then, various iterations of the ouabain-induced mania model have been established in both rats and mice [17,18,19]. Mechanistically, ICV ouabain has been shown to induce hyperactivation of the dopaminergic system in the rodent striatum by disrupting DA reuptake [19], stimulating tyrosine hydroxylase activity [20], and activating D2 dopamine receptors alongside Akt and ERK1/2 kinase signaling pathways [19]. Collectively, these data indicate that a primary mechanism by which ouabain modulates behavior is through the comprehensive alteration of dopaminergic signaling, encompassing changes in neurotransmitter flux, receptor activation, and the engagement of dopamine-dependent intracellular cascades.

Beyond mania-like phenotypes, direct administration of ouabain into the striatum has been shown to induce motor deficits reminiscent of those observed in patients with Rapid-onset Dystonia-Parkinsonism [21]. Similarly, chronic ICV delivery of ouabain at doses that are non-toxic upon single injection can also trigger motor impairments in mice [22]. Furthermore, the behavioral effects of ICV ouabain in rats have been associated with the induction of brain oxidative stress [23,24]. However, subsequent findings have introduced a degree of controversy; for instance, while antioxidants and BDNF successfully mitigate ouabain-induced oxidative stress, they do not always rescue mania-like behaviors—a result that partially contradicts earlier reports [25,26]. Conversely, agents such as the antioxidant genistein, melatonin, valproate, and lithium have been shown to reduce both oxidative stress and the associated behavioral abnormalities [27,28,29]. While the majority of research points toward a causal link between oxidative stress and the behavioral effects of CTS, a definitive conclusion requires further investigation into whether behavioral changes can persist in the absence of induced oxidative damage.

All the data presented above specifically concerns the effects of ouabain. While it could be argued that since all CTS serve as ligands for Na^+^,K^+^-ATPase, findings for ouabain may be extrapolated to the entire class, this assumption is oversimplified. Emerging evidence indicates that different CTS can elicit distinct physiological and signaling responses in neurons—including varying levels of neurotoxicity—despite inhibiting the same Na^+^,K^+^-ATPase pump and activating similar intracellular cascades [30,31,32]. These divergent effects are not limited to the CNS and have also been documented in other organs and cell types [33]. Intrahippocampal administration of very-low-dose ouabain (60 pg) stimulated ATII in the SON, produced natriuresis, 40 mmHg rise in BP, inhibition of sodium-pump in the renal medulla (19.6%) and aorta (25%), and caused a two-fold increase in renal marinobufagenin excretion [34].

Among endogenous mammalian CTS, marinobufagenin (MBG) is one of the most extensively characterized [35]. Elevated levels of endogenous MBG are clinically associated with salt-sensitive hypertension [36,37], chronic kidney disease [38], and preeclampsia [39]. In the CNS, MBG levels are notably increased in patients following traumatic brain injury [40,41]. Cellular models have further demonstrated that MBG enhances endothelial permeability, including within the cerebral vasculature, suggesting a potential role in compromising the integrity of the blood-brain barrier [42,43].

In animal models, systemic administration of MBG has been shown to suppress ethanol-seeking behavior [44]. Furthermore, in Dahl salt-sensitive rats, chronic systemic delivery via osmotic pumps induces cerebral vascular fibrosis, cognitive deficits, and hippocampal neurodegeneration [45]. However, to date, no direct comparison has been made between the effects of ICV administration of MBG and ouabain. By utilizing ICV administration, we can significantly diminish the influence of systemic factors and isolate the drug’s specific impact on the CNS. Consequently, we aimed to investigate the effects of ICV marinobufagenin in mice to provide a comparative analysis with previously established ouabain data, evaluating its influence on locomotor activity, anxiety, gait, monoamine metabolism across various brain structures, and the presence of oxidative stress.

## 2. Results

### 2.1. Impact of 14-Day ICV Administration of 1.5 μL of 100 μM Marinobufagenin on Locomotor Activity and Anxiety-like Behavior in Mice During a 20-min Open Field Test

**Figure 1:**
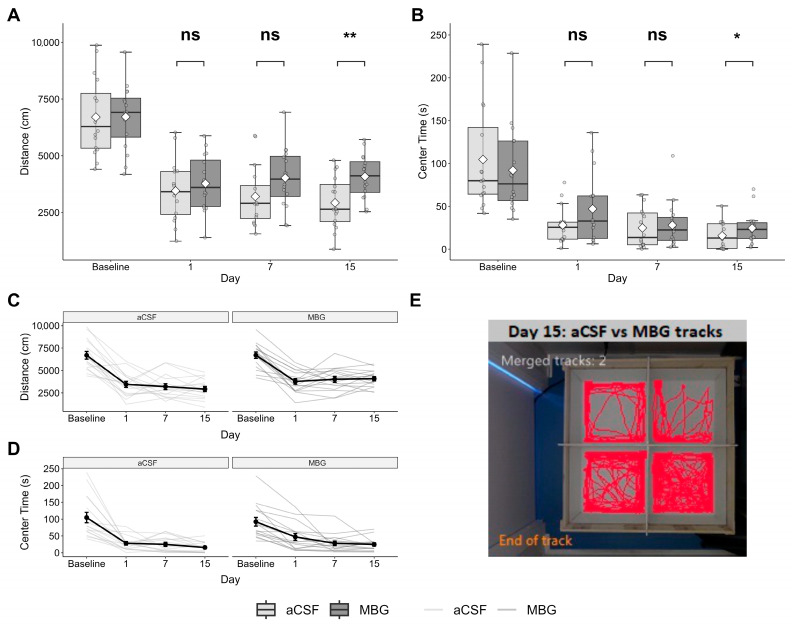
Longitudinal assessment of locomotor and anxiety-related behavior. (**A**) Total distance travelled in the open field during habituation and across testing days. (**B**) Time spent in the center of the open field during habituation and across testing days. Spaghetti plots showing the individual distance (**C**) and time spent in the center (**D**) changes across baseline and testing days in the MBG and aCSF groups. (**E**) Representative tracks of aCSF (top) and MBG (bottom) animals in the open field on day 15. In boxplots, horizontal lines represent medians, white diamonds indicate group means, and individual data points are shown as jittered circles. Statistical analysis was performed using linear mixed models with a baseline covariate and mouse ID as a random intercept. Asterisks indicate post-hoc Tukey-adjusted significance between groups (aCSF vs. MBG) at specific time points (* *p* < 0.05, ** *p* < 0.01; ns, not significant). *n* = 32 mice total (*n* = 16 per group). Detailed statistical summaries and sample sizes are provided in Table S1.

Prior to beginning ICV injections, animals were subjected to a 20-min habituation in the open field and subsequently split into two groups with equivalent baseline locomotor activity (Figure 1A). The control group (*n* = 16) received a daily ICV injection of 1.5 μL artificial cerebrospinal fluid (aCSF) for 14 days, while the marinobufagenin (MBG) group received 1.5 μL of 100 μM MBG. Locomotor performance was then assessed via 20-min open field tests at three distinct time points: immediately following the initial ICV injection, on day 7 (prior to the daily dose), and on day 15, 24 h after the final administration.

Locomotor activity was analyzed using a linear mixed-effects model (LMM), revealing a significant main effect of treatment (F1,29 = 5.39, *p* = 0.028). Baseline distance travelled during habituation did not differ significantly between groups (6705.22 ± 427.61 aCSF vs. 6717.84 ± 355.47 cm MBG, *p* = 0.982; Figure 1A). While groups exhibited comparable baseline-adjusted distance at day 1 (3456.88 ± 334.90 cm aCSF vs. 3778.46 ± 308.78 cm MBG, *p* = 0.46), a progressive divergence emerged over the treatment period. By day 15, MBG-treated mice displayed significantly greater total distance compared to aCSF controls (2928.71 ± 286.77 cm aCSF vs. 4087.02 ± 240.75 cm MBG, *p* = 0.009; Figure 1A). Notably, control animals exhibited a standard habituation-linked decline in activity across the 15-day period, whereas the MBG group maintained elevated locomotion (Figure 1C). Baseline distance travelled was not a significant predictor of these trajectories (*p* = 0.24).

Time spent in the center of the open field was analyzed using a generalized linear mixed model (GLMM). Baseline center time during habituation did not differ significantly between groups (29.38 ± 4.35 s aCSF vs. 33.45 ± 4.88 s MBG, *p* = 0.537; Figure 1B). Unlike locomotor distance, subsequent center exploration was significantly predicted by individual baseline performance (χ^2^ = 4.34, *p* = 0.037). While the global Treatment × Time interaction did not reach significance (*p* = 0.47), post hoc comparisons revealed a significant terminal divergence. On day 1, center time was comparable between groups (28.20 ± 5.21 s aCSF vs. 47.11 ± 10.05 s MBG, *p* = 0.24, Figure 1B). Both groups exhibited a significant time-dependent decrease in center exploration (χ^2^ = 11.25, *p* = 0.0036). However, by day 15, MBG-treated mice spent significantly more time in the center zone compared to aCSF controls (15.73 ± 3.87 s aCSF vs. 24.66 ± 4.78 s MBG, *p* = 0.038; Figure 1B,D,E). These results suggest that MBG treatment interfered with the decline in center exploration observed in control animals. Detailed statistical summaries are provided in Table S1.

### 2.2. Impact of 14-Day ICV Administration of 1.5 μL of 100 μM Marinobufagenin on Gait

**Figure 2:**
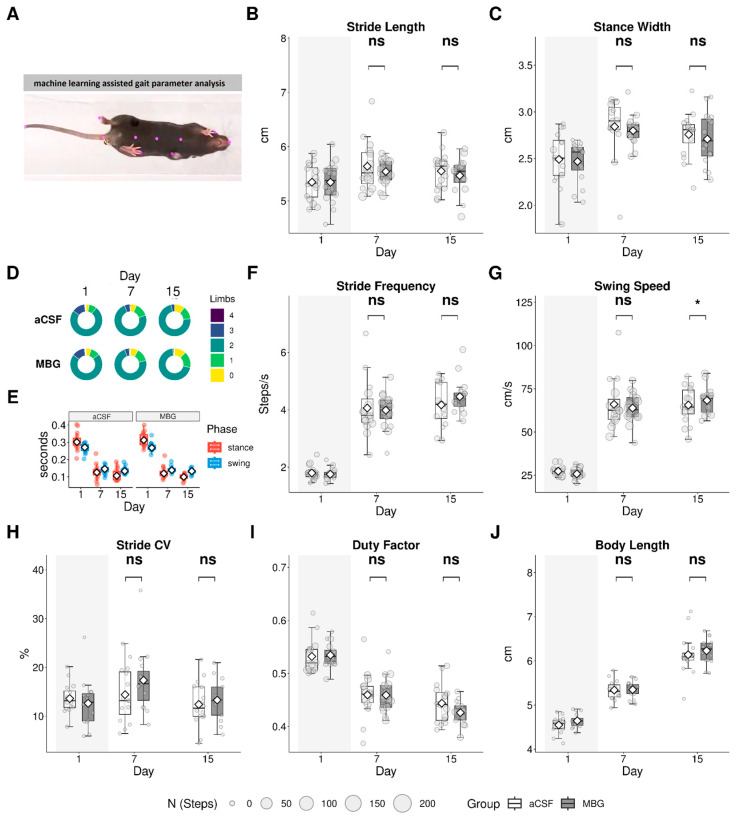
Spatiotemporal gait dynamics and locomotor coordination following chronic ICV MBG administration. (**A**) Representative video frame of mouse view from below used to determine gait parameters. (**B**) Stride length and (**C**) stance width trajectories across the 15-day experimental period. (**D**) Support configuration analysis showing the distribution of limb–ground contacts (0–4 limbs) and (**E**) phase duration breakdown illustrating the temporal balance between swing and stance phases. (**F**) Stride frequency (cadence) and swing speed (**G**) trajectories. (**H**) Stride coefficient of variation (CV), representing movement rhythmicity and locomotor stability. (**I**) Duty factor and (**J**) body length measurements. In all longitudinal plots, gray-shaded regions indicate the baseline covariate period (day 1). Horizontal lines represent medians, white diamonds represent mean values. Individual data points are shown as jittered circles, with size proportional to step-count reliability for each recording session. Statistical analysis was performed using weighted linear mixed models with day 1 values as a baseline covariate and mouse ID as a random intercept. Asterisks indicate post-hoc Tukey-adjusted significance between groups (aCSF vs. MBG) at specific time points (* *p* < 0.05; ns, not significant). *n* = 32 mice total (*n* = 16 per group). Detailed statistical summaries and sample sizes are provided in Table S1.

Gait kinetics and anatomical parameters were analyzed using weighted linear mixed-effects models (WLMM) to assess the impact of chronic ICV MBG administration. Overall, MBG treatment did not significantly alter the mechanical execution or fundamental spatiotemporal characteristics of gait compared to aCSF controls. Baseline assessments on day 1 confirmed no pre-existing differences between groups for any primary metric, including stride length and stance width (*p* > 0.05; Figure 2).

Throughout the longitudinal assessment, gait trajectories remained largely comparable between groups. At the midpoint of administration (day 7), primary indicators of locomotor coordination such as stride length (5.525 ± 0.075 cm MBG vs. 5.591 ± 0.079 cm aCSF, *p* = 0.546; Figure 2B) and stance width (2.782 ± 0.040 cm MBG vs. 2.873 ± 0.043 cm aCSF, *p* = 0.121; Figure 2C) exhibited no treatment-linked divergence. Similarly, other metrics remained stable, with no significant differences observed in stride frequency (Figure 2F), swing speed (Figure 2G). The weighted linear mixed model revealed a significant difference between aCSF and MBG in duty factor (F = 4.39, *p* = 0.0444) (Table S1), although this difference was not significant in post hoc after correcting for multiple comparisons (Figure 2I). Stride CV (16.785 ± 1.155% MBG vs. 14.660 ± 1.190% aCSF, *p* = 0.202; Figure 2H) and body length (5.287 ± 0.060 cm MBG vs. 5.344 ± 0.063 cm aCSF, *p* = 0.525; Figure 2J) also remained unchanged.

By the terminal assessment on Day 15, the fundamental gait pattern was preserved across both groups. MBG-treated animals exhibited a slight decrease in stance duration (0.114 ± 0.008 vs. 0.095 ± 0.006), *p* = 0.0448 (Figure 2E), an increase in swing speed (0.114 ± 0.008 vs. 0.095 ± 0.006), *p* = 0.0441 (Figure 2G) and a slight downward trend in duty factor compared to controls, which did not reach statistical significance (0.421 ± 0.009 MBG vs. 0.446 ± 0.009 aCSF, *p* = 0.051; Figure 2I), showing that MBG-treated mice tended to move faster than aCSF. Temporal coordination was further confirmed by stable stride frequencies (4.566 ± 0.187 steps/s MBG vs. 4.075 ± 0.191 steps/s aCSF), *p* = 0.067 (Figure 2H). Qualitative analysis of support configurations (Figure 2D) and phase durations (Figure 2E) likewise demonstrated that the transition between limb–ground contacts and the swing-stance balance was maintained. These findings indicate that chronic ICV MBG does not induce postural instability or significant deficits in rodent gait. Detailed statistical summaries are provided in Table S1.

### 2.3. Effects of 14-Day ICV Administration of 1.5 μL of 100 μM Marinobufagenin on Monoamine and Metabolite Concentrations in Mouse Brain Tissue

Following the behavioral testing on day 15, the concentrations of dopamine (DA), norepinephrine (NE), 3,4-dihydroxyphenylacetic acid (DOPAC), homovanillic acid (HVA), 3-methoxytyramine (3-MT), serotonin (5-HT), and 5-hydroxyindoleacetic acid (5-HIAA) were quantified in various brain regions—specifically the striatum, prefrontal cortex (PFC), hippocampus, thalamus, brainstem, and cerebellum—of both control and MBG-treated animals.

**Figure 3:**
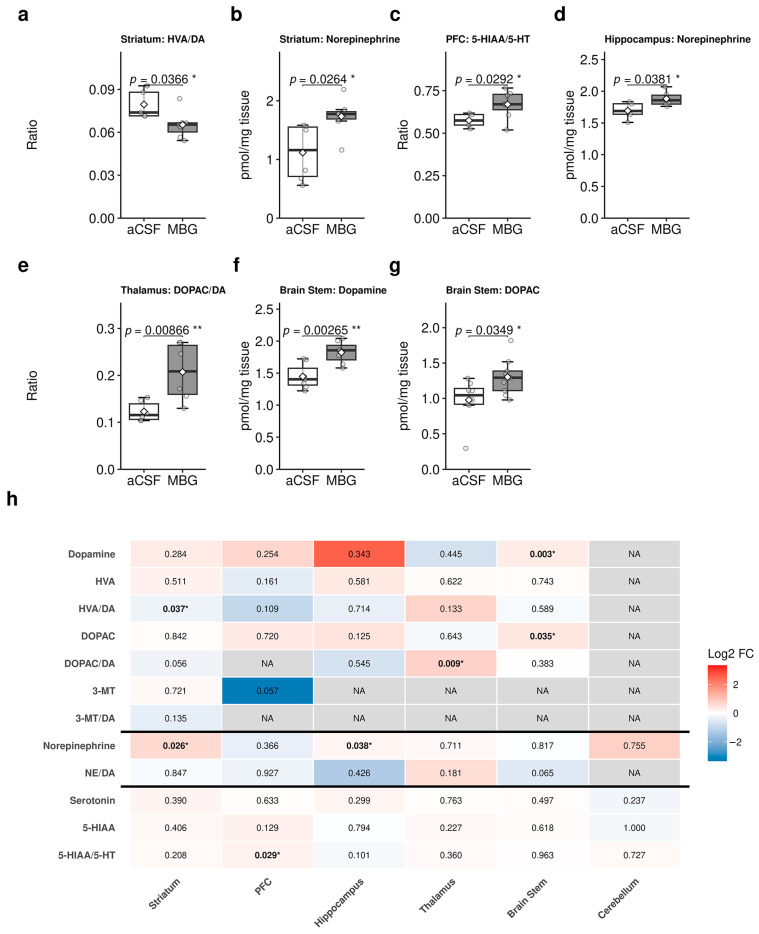
Regional neurochemical alterations following chronic ICV MBG administration. Concentrations of monoamines, their metabolites, and associated metabolic ratios in brain regions exhibiting significant alterations after 14 days of intracerebroventricular (ICV) 1.5 μL 100 μM marinobufagenin (MBG) administration. (**a**–**g**) Individual boxplots for significant findings across the striatum, brainstem, thalamus, prefrontal cortex (PFC), and hippocampus. Data are expressed as pmol/mg tissue and presented as boxplots with individual data points overlaid. Mean values are indicated by diamonds. (**h**) Comprehensive heatmap of Log2 fold-changes across all measured structures and compounds. Red indicates an increase and blue indicates a decrease relative to the aCSF control group. Grey (NA) cells denote comparisons with insufficient sample size (*n* < 3). Statistical significance: * *p* < 0.05; ** *p* < 0.01. Detailed statistical summaries and sample sizes are provided in Table A1.

In the striatum, chronic MBG treatment resulted in a 1.55-fold elevation in NE levels (1.73 ± 0.12 vs. 1.12 ± 0.20 pmol/mg; *p* = 0.026; Figure 3a, Table A1) and a significant decrease in the HVA/DA ratio (0.065 ± 0.004 vs. 0.080 ± 0.005; *p* = 0.037; Figure 3b, Table A1), suggesting a reduction in DA turnover in this region.

In the prefrontal cortex, MBG administration led to a 1.16-fold increase in the 5-HIAA/5-HT ratio (0.67 ± 0.03 vs. 0.58 ± 0.02; *p* = 0.029; Figure 3c, Table A1). Furthermore, NE levels in the hippocampus were significantly increased in MBG-treated animals compared to controls (1.88 ± 0.05 vs. 1.69 ± 0.06 pmol/mg; *p* = 0.038; Figure 3d, Table A1). A comprehensive heatmap summarizing regional metabolic shifts is provided in Figure 3h.

In the thalamus, the DOPAC/DA ratio was significantly elevated in the MBG group, increasing 1.8-fold compared to controls (0.209 [0.160–0.264] vs. 0.115 [0.106–0.139]; *p* = 0.009; Figure 3e, Table A1). In the brain stem, there was a 1.26-fold increase in DA levels (1.82 ± 0.07 vs. 1.45 ± 0.07 pmol/mg; *p* = 0.003; Figure 3f, Table A1) accompanied by a 1.33-fold increase in its metabolite, DOPAC (1.30 ± 0.09 vs. 0.98 ± 0.11 pmol/mg; *p* = 0.035; Figure 3g, Table A1).

A comprehensive heatmap summarizing all other measurements, including non-significant ones, is provided in Figure 3h. Collectively, these data demonstrate that 14-day ICV administration of 100 μM MBG (1.5 μL/day) induces widespread, region-specific dysregulation of the dopaminergic, noradrenergic, and serotonergic systems across the brain.

### 2.4. The Effects of 14-Day ICV Administration of 1.5 μL of 100 μM Marinobufagenin on Monoamine Oxidase B, Catalase, and Superoxide Dismutase Activities, and Malondialdehyde Levels in Mouse Brain Tissue

Following the behavioral assessments on day 15, the activity of monoamine oxidase B (MAO-B), superoxide dismutase (SOD), and catalase, as well as the concentration of malondialdehyde (MDA), were quantified in the striatum and PFC of animals from both the control (*n* = 8) and MBG-treated (*n* = 6) groups.

**Figure 4:**
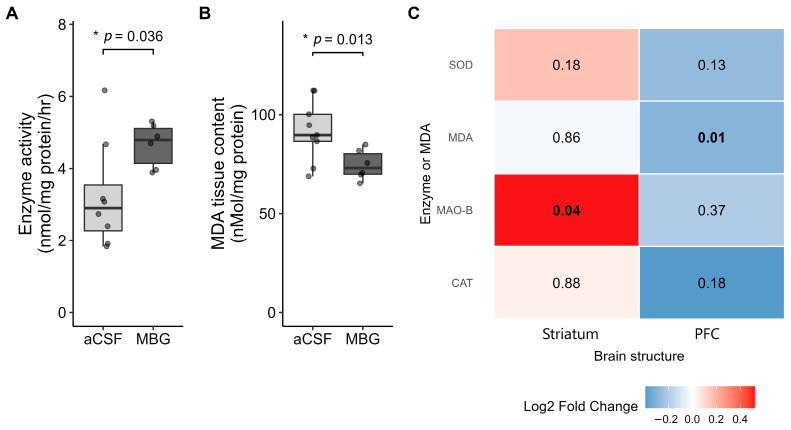
Impact of 14-day ICV administration of 1.5 μL of 100 μM marinobufagenin (MBG) on biochemical parameters in mouse striatal and prefrontal cortex (PFC) tissue. (**A**) Changes in MAO-B activity in the striatum. (**B**) Changes in MDA levels in the PFC. (**C**) Summary heatmap illustrating changes in SOD, catalase, and MAO-B activities, as well as MDA concentration in the striatum and PFC; the color gradient reflects the magnitude of change. Data are presented as mean ± SEM, * *p* < 0.05. Detailed statistical summaries and sample sizes are provided in Table A2.

As illustrated in Figure 4A,C, the 14-day ICV administration of 1.5 μL of 100 μM MBG resulted in a 1.43-fold increase in MAO-B activity within the striatum (*p* = 0.036). No significant differences in MAO-B activity were observed in the PFC between the experimental groups. Furthermore, no statistically significant differences were detected in SOD or catalase activities in either the striatum or the PFC. MBG did not alter MDA levels in the striatum; however, it induced a 1.23-fold decrease in MDA concentration in the PFC relative to the control group (*p* = 0.01) (Figure 4B,C). Comprehensive measurement results are provided in Table A2.

## 3. Discussion

According to our findings, a single intracerebroventricular (ICV) administration of 1.5 μL of 100 μM marinobufagenin (MBG) failed to alter locomotor activity or the duration of time spent in the center of the open field. These metrics remained unchanged even after seven days of repeated administration. Such observations stand in marked contrast to the effects of ICV ouabain administration in mice. Specifically, ICV injection of 1.5 μL of 50 μM ouabain has been shown to induce an immediate increase in locomotor activity and a concurrent reduction in anxiety-like behavior [19]. While some studies utilizing ICV ouabain in rats report the emergence of mania-like behavior several days post-injection [18], the divergent effects observed here—given the identical experimental design and animal model—underscore a fundamental difference between the influence of the cardiotonic steroids (CTS) MBG and ouabain on locomotor activity. Significant differences between the control and MBG-treated groups emerged only on the 15th day of the experiment, following two weeks of daily ICV administrations. Mice receiving chronic MBG treatment exhibited increased distance traveled in the open field and more time spent within the central zone. An increase in time spent in the center of the open field is traditionally interpreted as a sign of reduced anxiety [46].

Longitudinal gait assessment revealed a shift in spatiotemporal parameters observed in both experimental groups, likely reflecting a progressive habituation to the test. Across the 15-day period, all animals exhibited increasing swing speed. This adaptation was most clearly reflected in the duty factor, which decreased from a stable walking/slow trot baseline (approx 0.50) toward values nearing 0.40 by Day 15, and a concomitant trend toward increased stride frequency. Mechanistically, these changes signify a transition from a standard trot gait—defined by a duty factor of 0.5 or above and the absence of a defined aerial phase—to a fast trot. Chronic ICV MBG administration caused the animals to move even faster than the control group, which aligns with the increased locomotor activity observed in the open field. The gait cycle, coordination of limb support configurations, and the ability to adopt more efficient locomotor patterns remained preserved in MBG-treated animals. This suggests an absence of pronounced neurodegenerative changes within the basal ganglia, which regulate motor function. Specifically, the lack of changes in stride length indicates the absence of gait impairment. The lack of motor impairment following chronic MBG administration stands in contrast to the effects of chronic ouabain exposure, which has been shown to induce significant motor deficits, including increased traversal time and higher error rates in the circular beam and ladder tests [22].

Several hypotheses may account for why MBG elicits behavioral effects distinct from those of ouabain. First, MBG may exhibit inferior penetration into specific brain regions due to the differing polarities of CTS molecules [47]. Second, MBG may possess a significantly higher inhibition constant (K_i_) for Na^+^,K^+^-ATPase isoforms in the murine brain compared to ouabain. While such a disparity has been demonstrated in human renal microsomal fractions [48], these findings cannot be directly extrapolated to the mouse brain. This is primarily because the kidneys exclusively express the α1 isoform of Na^+^,K^+^-ATPase [49], and rodent Na^+^,K^+^-ATPase—particularly the α1 isoform—is notably less sensitive to CTS inhibition than its human counterpart [50].

Alternatively, different CTS may differentially modulate the activation of intracellular signaling cascades, leading to distinct biochemical outcomes [30,31,32]. It is also plausible that these three explanations are not mutually exclusive and contribute collectively to the observed differences in central nervous system response. Verifying the first two hypotheses remains beyond the scope of the present study and warrants further investigation.

To evaluate the third hypothesis, we compared the ratios of monoamines and their metabolites across brain structures following chronic ICV administration. Analysis of dopamine (DA) and its metabolites revealed a significant reduction in the HVA/DA ratio within the striatum of the MBG-treated group compared to aCSF. A decline in the ratio of DA metabolites to DA is a characteristic hallmark of dopamine transporter (DAT) inhibitors, such as cocaine [51] and GBR 12909 [52]. Consequently, our data suggest that chronic ICV administration of MBG may suppress DA reuptake within the striatum.

Neuronal culture models have previously demonstrated that 0.5–10 nM ouabain reduces Ca^2+^ influx through glutamatergic NMDA receptors (NMDAR) [53,54], thereby potentially inhibiting glutamatergic transmission. In vivo, NMDAR inhibitors, most notably MK-801, are known to induce hyperlocomotion [55]. Furthermore, NMDAR blockade via MK-801 leads to an increase in DA release and a concomitant rise in the tissue ratio of DA metabolites (DOPAC, HVA) to DA [56,57]. Studies measuring extracellular concentrations have similarly shown that ouabain triggers DA release in the striatum [13].

Following an acute ICV injection of ouabain, which induces hyperlocomotion, the HVA/DA ratio in the striatal tissue increases [19]. This observation stands in direct opposition to the effect identified for MBG—a reduction in the HVA/DA ratio. Moreover, in vitro evidence indicates that, unlike ouabain, MBG does not influence NMDAR activation [31], further distinguishing the neurochemical profiles of these two steroids.

Based on the synthesis of these data, it can be inferred that both MBG and ouabain enhance dopaminergic transmission, manifested as animal hyperactivity. However, unlike ouabain, the administration of MBG does not appear to trigger an increase in DA release—a distinction likely attributable to its lack of influence on glutamatergic transmission. This divergence in underlying mechanisms may also explain why the behavioral effects of MBG manifest only after 14 days of administration, whereas the effects of ouabain are immediate [19].

In the thalamus of MBG-treated mice, we observed a metabolic profile directly inverse to that seen in the striatum, characterized by an increase in the DOPAC/DA ratio. It should be noted that DA levels in the thalamus are an order of magnitude lower than those in the striatum. In the thalamic region, DA functions primarily as a neuromodulator, and dopaminergic innervation in rodents is significantly less developed compared to primates [58,59]. These dopaminergic projections originate from various brain regions, including the brainstem nuclei [59]. Our results indicate that MBG increases DA levels in the brainstem, likely through the stimulation of its synthesis, a phenomenon previously documented for ouabain [20]. Consequently, the elevated levels of DA and its metabolites in both the brainstem and thalamus following chronic ICV MBG administration may be linked to upregulated DA synthesis. Nevertheless, confirming this hypothesis requires further investigation, specifically focusing on the influence of MBG on DA release and reuptake kinetics across these distinct murine brain structures.

Changes in serotonergic transmission likely contribute to the behavioral phenotypes observed following CTS administration. An elevated 5-HIAA/5-HT ratio observed in the prefrontal cortex (PFC) of MBG-treated mice may indicate increased serotonin turnover, a phenomenon previously reported following chronic fluoxetine administration in mice [60]. Such enhanced turnover potentially underlies the reduced anxiety-like behavior observed in the MBG-treated group, as increased serotonergic metabolism in the PFC is established as an anxiolytic marker [61,62]. Notably, this effect parallels observations from studies utilizing 14-day osmotic pump delivery of ouabain [63], suggesting that, unlike their divergent effects on dopaminergic transmission, MBG and ouabain exert comparable influences on the serotonergic system.

The present study also demonstrates that chronic MBG administration leads to increased norepinephrine (NE) concentrations within the striatal tissue. NE is recognized for its potent neuroprotective properties [64], and the degeneration of noradrenergic neurons is a hallmark of neurodegenerative progression. In our experimental model, the striatal NE increase may result from upregulated DA synthesis, which serves as a precursor for NE production [65]. While a direct causal link between elevated NE and the observed behavioral shifts remains to be fully elucidated, this noradrenergic surge may correlate with the absence of motor deficits and the reduction in malondialdehyde (MDA) levels noted in this work. These findings are consistent with the neuroprotective effects of MBG described elsewhere [64] and align with the known capacity of NE to preserve dopaminergic neurons from degenerative processes [64].

Assessment of signs of oxidative stress development showed that in contrast to the effects reported for ouabain [23], MBG did not alter the activity of superoxide dismutase (SOD) or catalase, nor did it induce an accumulation of MDA. On the contrary, MDA levels were significantly reduced within the prefrontal cortex (PFC). Given that standard agents used to induce cerebral oxidative stress, such as rotenone and MPTP, typically elevate MDA levels and perturb antioxidant enzyme activity [66,67], our data suggest that the hyperlocomotion induced by CTS is not necessarily contingent upon the development of oxidative stress. The observed increase in striatal MAO-B activity is likely a compensatory response to the reduction in DA reuptake induced by MBG. Supporting this, studies in DAT knockout mice have demonstrated that MAO-B inhibition leads to a significant decrease in DA elimination [68].

Furthermore, while ouabain has previously demonstrated neuroprotective efficacy in animal models of neuroinflammation [69], MBG appears to actively mitigate oxidative damage within brain tissue. This aligns with existing literature, such as findings that MBG effectively reduces amyloid precursor protein (APP) levels and neuroinflammatory markers in aged mice belonging to an Alzheimer’s disease model [70].

## 4. Materials and Methods

### 4.1. Animals

Adult C57/Black male mice aged 4–6 months (32 mice) provided by the Saint Petersburg State University vivarium were used in this study. Animals were housed in individually ventilated cages at a temperature of 22 ± 1 °C, 50–70% relative humidity and 12 h light/dark cycle (light from 8 a.m. to 8 p.m.), with food and water available ad libitum. All studies were conducted in accordance with the principles of biomedical ethics as outlined in the 1964 Declaration of Helsinki and its later amendments. They were also approved by the Ethics Committee for Animal Experiments of Saint Petersburg State University (St. Petersburg, Russia; protocol no. 131-03-3 from 3 March 2026).

### 4.2. Surgery

Animals were anesthetized with isoflurane and secured in a stereotaxic manipulator. The scalp was shaved, disinfected with iodine, and a longitudinal incision was made. The skull was exposed and cleared of connective tissue. A craniotomy (~0.8 mm in diameter) was performed using a dental bit at the following stereotaxic coordinates: AP = −0.5 mm and ML = 1.0 mm relative to bregma. A guide cannula was inserted into the lateral ventricle to a depth of 2 mm and secured with an ultraviolet light-cured dental composite. To maintain patency, a dummy cannula was inserted into the guide cannula. Guide and dummy cannula were prepared in-lab in accordance with a previously published protocol [71]. The animals were allowed to recover for 4–5 days before further manipulations.

### 4.3. Experimental Design

A total of 32 cannulated mice were divided into two experimental groups (*n* = 16 per group) with balanced baseline locomotor activity as determined by an open field test. The control group received a daily unilateral intracerebroventricular (ICV) injection of 1.5 µL artificial cerebrospinal fluid (aCSF, 125 mM NaCl, 26 mM NaHCO_3_, 4 mM KCl, 1.25 mM NaH_2_PO_4_, 2 mM CaCl_2_, 2 mM MgCl_2_, 25 mM glucose) at a rate of 0.75 µL/min for 14 consecutive days. The experimental (MBG) group received a daily ICV injection of 100 µM marinobufagenin (Cayman Chemical, Ann Arbor, MI, USA) in aCSF using the same volume and flow rate. Behavioral assessments were conducted at three time points: day 1 (immediately following administration), day 7 (prior to administration), and day 15 (24 h after the final dose). Following the day 15 assessment, animals were euthanized via decapitation. Brain regions—including the prefrontal cortex, striatum, hippocampus, thalamus, cerebellum, and brainstem—were rapidly dissected on ice and flash-frozen in liquid nitrogen for storage. In the brain tissue of 8 mice from the control group and 10 mice from the MBG group, the levels of monoamines and their metabolites were measured. Additionally, in the brain tissue of 8 mice from the control group and 6 mice from the MBG group, measurements were taken for monoamine oxidase-B (MAO-B) activity, catalase, superoxide dismutase (SOD), and malondialdehyde (MDA) content.

### 4.4. Open Field Test for Locomotor Activity and Anxiety Assessment

Locomotor activity (total distance traveled, cm) and anxiety-like behavior (time spent in the center, s) were assessed longitudinally across four time points: habituation (day 0) and three treatment sessions (days 1, 7, and 15). The apparatus (four square 40 cm × 40 cm boxes) was divided into two zones: the center zone (20 × 20) and the “wall” zone for each box. Each mouse was placed individually in the center of the arena and a 20 min habituation in the open field was performed. Testing was performed for 20 min as well. Animal behavior was monitored by the video tracking system (EthoVision 11.5 XT video tracking software, Noldus), using a video camera placed above the boxes in a uniformly lit room.

### 4.5. Gait Parameter Assessment

#### 4.5.1. Video Acquisition and Experimental Setup

All motor impairment tests were conducted in a custom apparatus designed for synchronous multi-view video recording from below, left, and right perspectives based on a description previously published by a different research group [72]. The setup consisted of a white-painted enclosed box equipped with two mirrors, bottom-mounted LED lighting, and a video camera. Animals traversed a 120 cm path from start to finish. Videos were captured using a GoPro Hero 8 camera positioned 70 cm from the center of the testing area, recording in 2.7k resolution wide-angle mode at 60 frames per second with a 1/480 shutter speed. A transparent tunnel constructed out of transparent acrylic (7.5 cm wide, 15 cm tall, and ~120 cm long) was used. Animals underwent four days of pre-training to acclimatize to the tests. During training sessions, mice were placed at the test start point and allowed to move toward their home cage at the opposite end. Animals hesitating for more than 30 s were gently encouraged to proceed by touching the hind legs. Following test completion, mice remained in their home cage for 3–5 min before subsequent trials. Each animal completed three runs per test daily.

#### 4.5.2. Markerless Pose Estimation Using Lightning Pose

Videos of mice traversing the runway were pre-processed using a Python 3.10 script applying OpenCV contour detection to automatically identify and crop the mouse from both lateral and bottom views. Cropped views were then stitched into a single composite video (700 × 900 pixels). Lightning Pose v. 1.6.1 [73] was used to train the model used for markerless pose estimation. An experienced observer marked ~1200 frames selected using k-means clustering from 200 videos featuring 35 C57/Black mice and 10 CBA mice. Fourteen body parts were labeled in each frame (nose, left front third toe tip, right front third toe tip, wrist, shoulder, elbow, left hind limb third toe tip, right hind limb third toe tip, metatarsus, ankle, knee, iliac crest, hip, base of the tail, tip of the tail) were marked in profile, and 12 body parts (head, right front limb, left front limb, front limb girdle, right hind limb, right hind metatarsus, left hind limb, left hind metatarsus, hind limb girdle, middle of the body, base of the tail, end of the tail) from below. The marked frames were randomly divided into training and test samples (95%/5%). To train the model, we used the ResNet 50 model pre-trained on the AP-10k dataset [74]. The frames were reduced to 384 by 512 pixels for training while maintaining the aspect ratio. The remaining configuration parameters were not changed. Training was carried out for 600 epochs in semi-supervised mode with contextual corrections for pose, time, and three views, with a final testing error of 12.745 pixels. A cutoff of 0.9 certainty was used for analysis to exclude unreliable coordinates. Coordinates for all body parts estimated by the model were exported in CSV format, and gait parameter calculations were analyzed in the RStudio v. 2024.12.1+563 environment. Gait parameters were derived from the normalized anteroposterior (x-axis) paw trajectories. Only the bottom view was used for analysis in this study.

#### 4.5.3. Calculation of Gait Parameters

We used automated signal processing to isolate rhythmic locomotor periods from non-locomotor or poor-quality movement using a global stationary mask and hybrid anchored spectral logic. Stationary periods were identified via the standard deviation (SD) of paw x-coordinates within a 0.25-s sliding window (SD < 0.4); any segment where at least one limb was stationary was flagged for exclusion. For the remaining movement, gait quality was assessed using a sliding window (25 frames, ~0.42 s) where a score was calculated as the product of the maximum power within the locomotor frequency range (2–20 Hz) and the relative power (SNR). Windows were hierarchically filtered: those with high SD (>20) were automatically retained via a high-amplitude pass to capture significant gait alterations, while remaining windows were excluded if they fell below a global failure threshold or failed an SNR leniency check (SNR < 0.30). The final analysis was restricted to frames not contained within the combined stationary or poor-quality masks, with algorithm performance verified via composite diagnostic plots to distinguish genuine motor impairment from non-gait artifacts.

Swing and stance phases were automatically identified from the anteroposterior (x-axis) paw trajectory. The first and second derivatives of position were calculated to determine velocity and acceleration. Local maxima (peaks) and minima (troughs) in the signal were identified to capture motion extremes. A rule-based classifier assigned each frame a phase: sustained positive velocity relative to the body center defined swing, sustained negative velocity defined stance, and acceleration sign resolved low-velocity periods. Step cycle starts were defined by the stance-to-swing transition. Continuous locomotion sequences were isolated by segmenting data at inactivity periods > 1 s. Sequences with fewer than 3 complete cycles were discarded. Finally, each step was validated against minimum duration and length thresholds to remove artifacts. The accuracy of the step phase detection was confirmed for all trials by visually inspecting plots of the raw paw oscillations overlaid with their corresponding swing and stance phase assignments.

All parameters were calculated only for complete steps within continuous walking segments, excluding the first and last steps of each segment to ensure the analysis of stable, representative locomotion. Stride length (cm) was calculated as the Euclidean distance between a paw’s position at the start and end of successive stance phases, while stance width (cm) was determined for the hindlimbs by calculating the lateral (y-axis) distance between paired paws at the midpoint of each step cycle. Body length (cm) was defined as the Euclidean distance between the chest/shoulder center and pelvic center tracking points for each video frame, with all pixel coordinates converted to centimeters using a verified conversion factor of 21 pixels/cm. Stride frequency (steps/s) was calculated as the inverse of the stride duration, and duty factor was computed as the ratio of stance duration to total stride duration, representing the fraction of the gait cycle the limb remains in contact with the ground. Stride CV (%) was calculated as the coefficient of variation (100 × Mean SD) of stride duration to quantify gait rhythmicity and locomotor stability. Finally, support configurations were categorized by identifying the number of limbs in contact with the ground (0–4 limbs) at every frame and expressed as a percentage of the total gait cycle, while phase durations were isolated by defining the stance phase (initial contact to toe-off) and swing phase (toe-off to subsequent initial contact) in seconds.

### 4.6. Monoamine Level Evaluation Using High-Performance Liquid Chromatography with Electrochemical Detection

Tissues were homogenized in 20 volumes of extraction medium (0.1 N HClO_4_ with 0.25 nmol/mL DBS (3,4-dihydroxybenzylamine) added as an internal standard) using a glass/Teflon pestle homogenizer (0.2 mm), Schuett Homgen plus (SchuetBiotec GmbH, Göttingen Germany), at a pestle rotation speed of 3000 rpm in an ice water bath. Samples were centrifuged at 10,000× *g* for 15 min (t = 4 °C).

Monoamine and metabolite concentrations were quantified in samples via high-performance liquid chromatography with electrochemical detection (HPLC-ED). The analytical system consisted of a Beckman Coulter System Gold chromatograph utilizing a Rheodyne 7125 injector (Sigma-Aldrich, St. Louis, MO, USA) (20 μL loop). Analytes were separated on a Nucleodur C18 Gravity column (4.6 × 250 mm, 5 μm; Macherey-Nagel, Düren, Germany) maintained at a flow rate of 1 mL/min (200 atm) via a System Gold 125 pump. The mobile phase was composed of a 0.1 M citrate-phosphate buffer (pH 3.0) supplemented with 1.1 mM octanesulfonic acid, 0.1 mM EDTA, and 9% acetonitrile. Detection was achieved using a RECIPE EC3000 electrochemical detector (Sputnik ClinLab ECD cell, RECIPE Chemicals + Instruments GmbH, München-Moosach, Germany) featuring a glassy carbon working electrode (+0.85 V) and an Ag/AgCl reference electrode. Data acquisition and peak integration were managed through MULTICHROM 1.5 software. Quantification was performed using an internal standard method, calibrated against a 0.25 nmol/mL standard mixture of all target analytes.

Quantification of monoamines (Norepinephrine, Dopamine, Serotonin) and their metabolites (DOPAC, HVA, 3-MT, 5-HIAA) was performed across six distinct brain structures: striatum, prefrontal cortex, hippocampus, thalamus, brainstem, and cerebellum.

Raw HPLC data (pmol/mL) were merged with sample preparation records and normalized to tissue weight, with final concentrations expressed as pmol/mg of tissue. To assess neurotransmitter utilization and metabolism, the following metabolic ratios were calculated for each sample: 5-HIAA/5-HT, HVA/DA, DOPAC/DA, 3-MT/DA, and NE/DA. Samples with missing values or those falling outside physiologically plausible ranges were excluded from the final analysis.

### 4.7. Determination of Enzyme Activity and MDA Content

#### 4.7.1. Determination of Cu/Zn-Superoxide Dismutase Activity

Cu/Zn-SOD activity was measured in 10% striatal and frontal cortex homogenates using a modified method [75], adapted for 96-well microplates. The assay is based on the inhibition of nitroblue tetrazolium (NBT) reduction by superoxide radicals generated through phenazine methosulfate (PMS) auto-oxidation. The reaction mixture contained 125 µL of 2 mM Na-pyrophosphate buffer with 2 mM Na-EDTA (pH 8.3), 17 µL of 0.5 mM NBT, 17 µL of 1.4 mM NADH, and 5 µL of the post-mitochondrial fraction. Following a 1-min pre-incubation at 37 °C, the reaction was initiated by adding 17 µL of 22.2 µM PMS. Absorbance was monitored at λ = 540 nm using a Synergy H4 plate reader (Agilent BioTek, Santa Clara, CA, USA). Blank samples were prepared by omitting PMS. SOD activity was defined as the amount of enzyme required to inhibit NBT reduction by 50% and expressed as Units/min/mg of protein, calculated as: A = (A_op_ − A_0_)/T_peak_. Where A_op_ is the peak optical density, A_0_ is the blank, and Tpeak is the time to reach maximum absorbance.

#### 4.7.2. Catalase Activity Assay

Catalase (CAT) activity was measured in 10% striatal and frontal cortex homogenates according to the method described by [76], adapted for 96-well microplates. The assay evaluates enzyme activity based on the degradation rate of hydrogen peroxide (H_2_O_2_) in the incubation medium. The remaining H_2_O_2_ concentration was determined by its reaction with ammonium molybdate, which forms a stable colored complex. Absorbance was measured at λ = 410 nm using a Synergy H4 plate reader (BioTek, USA). CAT activity (A) was calculated using the following formula: A = T(OD_0_ − OD_op_) × K Where: A is CAT activity (μmol H_2_O_2_/mg protein / min); OD_op_ is absorbance of the experimental sample; OD_0_ is absorbance of the blank (control) sample; K is the molar extinction coefficient for conversion to μmol H_2_O_2_; T is the incubation time (min).

#### 4.7.3. Monoamine Oxidase-B (MAO-B) Activity Assay

The mitochondrial fraction was isolated from striatal and frontal cortex homogenates using differential centrifugation. Tissue samples were homogenized in 20 volumes of ice-cold 0.32 M sucrose. The homogenate was first centrifuged at 2000× *g* for 10 min to remove cellular debris. The resulting supernatant was collected and centrifuged at 20,000× *g* for 20 min. To remove myelin, the pellet was washed with 0.2 M K,Na-phosphate buffer (pH 7.6) and centrifuged again at 20,000× *g* for 20 min. The final mitochondrial pellet was resuspended in the phosphate buffer (matching the initial sucrose volume) and stored at −80 °C.

MAO-B activity was determined according to a modified method by [77], based on the oxidative deamination of benzylamine. The reaction mixture consisted of 50 µL of the mitochondrial fraction, 400 µL of 0.2 M K,Na-phosphate buffer (pH 7.6), and 50 µL of 80 mM benzylamine. Blank samples were prepared by omitting the mitochondrial homogenate. After a 3-h incubation at 37 °C with continuous agitation, the reaction was terminated by adding 150 µL of 20% trichloroacetic acid (TCA). The reaction product, benzaldehyde, was extracted by adding 750 µL of n-hexane, followed by vigorous shaking and centrifugation at 3000× *g* for 10 min. The absorbance of the hexane layer was measured at λ = 242 nm using an Ultraspec 3300 Pro spectrophotometer (Amersham Biosciences, Amersham, England) in quartz cuvettes. MAO-B activity was expressed as nmol of benzaldehyde/mg protein/h, calculated as: A = T(OD_op_ − OD_0_) × K, where OD_op_ is the sample absorbance, OD_0_ is the blank, K is the molar extinction coefficient for benzaldehyde, and T is the incubation time in hours.

#### 4.7.4. Quantifying Malondialdehyde Levels

Malondialdehyde (MDA) levels in the striatum and frontal cortex were quantified as a marker of lipid peroxidation using a modified thiobarbituric acid reactive substances (TBARS) protocol based on [78,79], adapted for high-throughput 96-well microplate analysis. The assay is based on the reaction of MDA with thiobarbituric acid (TBA) under acidic conditions and high temperature to form a colored trimethine complex. Briefly, 20 μL of 10% brain tissue homogenate was combined with 150 μL of 20% acetic acid, 20 μL of 8.1% sodium dodecyl sulfate (SDS), 150 μL of 0.8% TBA, and 60 μL of distilled water. The mixture was incubated at 95 °C for 120 min using a TT-2 “Termit” dry bath incubator (DNA-Technology, Moscow, Russia). Following incubation, samples were cooled on ice and centrifuged at 3000× *g* for 10 min. The resulting supernatant was collected, and optical density was measured at λ = 535 nm and λ = 580 nm (background correction) using a Synergy H4 microplate reader (Agilent BioTek, Santa Clara, CA, USA). MDA concentration was calculated using the molar extinction coefficient of the MDA-TBA conjugate (1.56 × 105 M^−1^cm^−1^) and normalized to total protein content (nmol/mg protein) according to the formula: C = [(OD_sample_ − OD_blank_)/(ε × l)] × 109, where OD_sample_ and OD_blank_ represent the absorbance of the sample and the reagent blank, respectively.

### 4.8. Statistical Analysis

Behavioral data, including distance travelled and time in center, were analyzed using mixed-effects models to account for the longitudinal structure of the data and individual variability, with mouse ID included as a random intercept in all models. To control for initial behavioral variance, baseline performance (day 0) was included as a fixed covariate for the analysis of subsequent test days. Group differences at baseline were separately assessed using independent-samples *t*-tests. Total distance was analyzed using a linear mixed-effects model (LMM) via the lme4 (v. 2.0.1) and lmerTest (v. 3.2.1) packages. Fixed factors included treatment group, day (as a categorical factor), their interaction, and baseline distance. Anxiety-like behavior (center time) was modeled using a generalized linear mixed-effects model (GLMM) via the glmmTMB (v. 1.1.14) package. Due to the right-skewed nature of the duration data, a Gamma distribution with a log-link function was used. To handle potential floor effects, the model was fitted to (center_time + 0.1). Model validity, overdispersion, and residual distribution were confirmed using simulation-based diagnostics via the DHARMa (v. 0.4.7) package.

Longitudinal gait data were analyzed using weighted linear mixed-effects models (WLMM) via the lme4 (v. 2.0.1) and lmerTest (v. 3.2.1) packages in R. To control for inter-individual variance, baseline performance on day 1 was included as a fixed covariate. The models utilized treatment group, day categorical, their interaction, and baseline metrics as fixed factors, with mouse ID as a random intercept. Parameters that failed normality checks were analyzed using GLMMs on log-transformed data. To account for sampling reliability, observations were weighted by session-specific step counts. Significance was determined using Type III ANOVA with Satterthwaite’s approximation for WLMMs and back-transformed response ratios for GLMMs.

Normality and homoscedasticity were confirmed via Shapiro-Wilk tests and Q-Q plot inspection of residuals. Post-hoc group comparisons at specific time points were performed using estimated marginal means with Tukey’s HSD adjustment. Results are reported as means ± standard error of the mean (SEM).

Statistical comparisons between treatment groups (aCSF vs. MBG) for HPLC and enzyme activity data were conducted independently for each compound within each anatomical structure. Descriptive statistics are reported as mean ± SEM for normal data, and median ± interquartile range (IQR) for non-normal data. All statistical tests were two-tailed, and significance was defined as α = 0.05. The choice of statistical test was determined by group size and distributional assumptions: Normality was assessed using the Shapiro-Wilk test. An independent-samples t-test was employed when data followed a normal distribution (*p* > 0.05). For non-normally distributed data or small sample sizes (*n* < 3), the Wilcoxon rank-sum test was used. To visualize global neurochemical shifts, Log2 fold-change values were calculated as Log2(Mean MBG/Mean aCSF). A heatmap was generated to display these shifts across all structures and compounds. Significance levels (*p*-values) were overlaid on the heatmap, with significant differences (*p* < 0.05) highlighted in bold.

All data analysis and visualization were performed in R (v. 4.5.2) using the tidyverse suite (v. 2.0.0) for data manipulation. Statistical tests were implemented via the rstatix package (v. 0.7.3). Figures were generated using ggplot2 (v. 4.0.2) and ggpubr (v. 0.6.3), with multi-panel assemblies made using the patchwork (v. 1.3.2) package.

## 5. Conclusions

**Figure 5:**
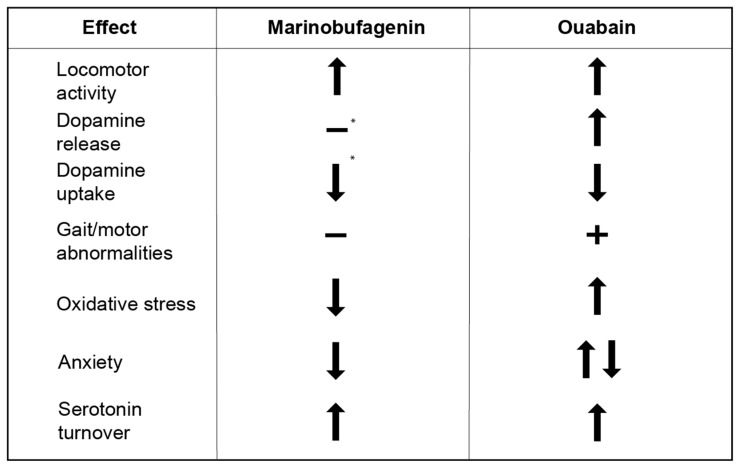
Comparison of the effects of marinobufagenin ICV administration in this study with the effects of ICV administration of ouabain in rodents [13,16,17,18,19,22,23,62,80] known from the literature data. *—inferred indirectly from the quantitative ratio of monoamines and their metabolites in brain tissue.

In contrast to ouabain, acute ICV administration of MBG does not alter locomotor activity or anxiety levels in mice. Following a chronic 14-day ICV regimen, MBG induces hyperlocomotion and anxiolysis; however, unlike ouabain, it does not precipitate motor impairment or oxidative stress in the striatum and PFC. Furthermore, MBG exerts a distinct influence on dopamine and its metabolites compared to ouabain, although both steroids similarly increase serotonin turnover in the PFC upon chronic administration (Figure 5).

## Data Availability

Data and analysis scripts used in this study are available in an OSF repository—https://osf.io/4rtsg/ accessed on 17 June 2026.

## Supplementary Materials

The following supporting information can be downloaded at https://www.mdpi.com/article/10.3390/ijms27135713/s1.

## Abbreviations

The following abbreviations are used in this manuscript:

3-MT	3-methoxytyramine
5-HIAA	5-hydroxyindoleacetic acid
5-HT	5-hydroxytriptamine
aCSF	Artificial cerebrospinal fluid
APP	Amyloid precursor protein
BDNF	Brain-derived neurotrophic factor
CAT	Catalase
CNS	Central nervous system
CTS	Cardiotonic steroids
CV	Coefficient of variation
DA	Dopamine
DAT	Dopamine active transporter
DBS	3,4-dihydroxybenzylamine
DOPAC	3,4-dihydroxyphenylacetic acid
EDTA	Ethylenediaminetetraacetic acid
ERK1/2	Extracellular Signal-Regulated Kinases 1 and 2
GLMM	Generalized linear mixed-effects model
HPLC	High-performance liquid chromatography
HPLC-ED	High-performance liquid chromatography with electrodetection
HSD	Honestly significant difference
HVA	Homovanillic acid
ICV	Intracerebroventricular
IQR	Interquartile range
Ki	Inhibition constant
LMM	Linear mixed-effects model
MAO-B	Monoamine oxidase B
MBG	Marinobufagenin
MDA	Malondialdehyde
MPTP	1-Methyl-4-phenyl-1,2,3,6-tetrahydropyridine
NBT	Nitroblue tetrazolium
NE	Norepinephrine
NMDA	*N*-Methyl-D-aspartate
NMDAR	*N*-Methyl-D-aspartate receptor
PFC	Prefrontal cortex
PMS	Phenazine methosulfate
SD	Standard deviation
SDS	Sodium dodecyl sulfate
SEM	Standard error of the mean
SOD	Superoxide dismutase
TBA	Thiobarbituric acid
WLMM	Weighted linear mixed-Effects model

## Appendix A

### Appendix A.1. Data Table for the Effect of 14-Day ICV Administration of 1.5 μL of 100 μM Marinobufagenin on the Amount of Monoamines, Their Metabolites, and Their Ratios on Day 15 of the Experiment in Mouse Brain Structures

**Table A1:** The effect of 14-day ICV administration of 1.5 μL of 100 μM marinobufagenin on the amount of monoamines, their metabolites, and their ratios on day 15 of the experiment in mouse brain structures. For the *t*-test, data are presented as mean ± SEM, and for the Wilcoxon test as median [IQR], with significant differences highlighted in bold.

Structure	Compound	Control vs. MBG (pmol/mg for Absolute Values)	Test	*p*
Hippocampus	NE	1.6947 ± 0.0589 vs. 1.8819 ± 0.0476	*t*-test	**0.0381**
Hippocampus	DOPAC	0.2515 ± 0.0261 vs. 0.3454 ± 0.0462	*t*-test	0.125
Hippocampus	HIAA	5.9356 ± 0.1775 vs. 5.7554 ± 0.6348	*t*-test	0.794
Hippocampus	HVA	0.4079 ± 0.1544 vs. 0.5336 ± 0.1557	*t*-test	0.581
Hippocampus	5-HT	4.5490 ± 0.2583 vs. 5.0496 ± 0.3790	*t*-test	0.299
Hippocampus	DA	0.2506 [0.1771–0.3923] vs. 1.0590 [0.3586–2.5754]	Wilcoxon	0.343
Hippocampus	HIAA_5-HT_ratio	1.2713 [1.1830–1.4119] vs. 1.0774 [1.0475–1.1096]	Wilcoxon	0.101
Hippocampus	HVA_DA_ratio	1.1365 ± 0.3989 vs. 0.8631 ± 0.5796	*t*-test	0.714
Hippocampus	DOPAC_DA_ratio	1.5596 ± 0.6692 vs. 0.9684 ± 0.6116	*t*-test	0.545
Hippocampus	NE_DA_ratio	10.4829 ± 6.1231 vs. 4.2907 ± 2.5094	*t*-test	0.426
Hippocampus	3-MT	0.2095 [0.1981–0.6791] vs. 0.1239 [0.1239–0.1239]	Wilcoxon	0.5
PFC	NE	1.6672 ± 0.2787 vs. 1.2815 ± 0.3026	*t*-test	0.366
PFC	DOPAC	0.3180 ± 0.2194 vs. 0.4126 ± 0.0935	*t*-test	0.72
PFC	HVA	0.8627 ± 0.1090 vs. 0.6349 ± 0.1077	*t*-test	0.161
PFC	5-HT	4.2426 ± 0.1875 vs. 4.3812 ± 0.2123	*t*-test	0.633
PFC	DA	0.7653 ± 0.2169 vs. 1.2336 ± 0.3169	*t*-test	0.254
PFC	HVA_DA_ratio	1.2187 [0.6982–2.0100] vs. 0.4531 [0.3494–0.6317]	Wilcoxon	0.109
PFC	DOPAC_DA_ratio	0.1435 ± 0.1390 vs. 0.6835 ± 0.2537	*t*-test	0.136
PFC	NE_DA_ratio	1.7584 [1.1213–3.2530] vs. 1.7953 [0.4173–2.1996]	Wilcoxon	0.927
PFC	HIAA	2.5269 ± 0.1564 vs. 2.9727 ± 0.2204	*t*-test	0.129
PFC	3-MT	0.3911 [0.1828–0.8129] vs. 0.0472 [0.0295–0.0776]	Wilcoxon	0.0571
PFC	HIAA_5-HT_ratio	0.5752 ± 0.0176 vs. 0.6697 ± 0.0319	*t*-test	**0.0292**
Striatum	NE	1.1174 ± 0.1970 vs. 1.7345 ± 0.1158	*t*-test	**0.0264**
Striatum	HIAA	6.1208 ± 0.5145 vs. 6.6921 ± 0.4113	*t*-test	0.406
Striatum	HVA	2.4295 ± 0.4219 vs. 2.8661 ± 0.4846	*t*-test	0.511
Striatum	5-HT	5.0970 ± 0.6585 vs. 5.7882 ± 0.3957	*t*-test	0.39
Striatum	HIAA_5-HT_ratio	1.0849 ± 0.0368 vs. 1.1636 ± 0.0459	*t*-test	0.208
Striatum	DOPAC	3.4969 ± 0.5131 vs. 3.6202 ± 0.2983	*t*-test	0.842
Striatum	DA	34.1594 ± 5.8863 vs. 42.9369 ± 4.9560	*t*-test	0.284
Striatum	HVA_DA_ratio	0.0795 ± 0.0045 vs. 0.0652 ± 0.0036	*t*-test	**0.0366**
Striatum	DOPAC_DA_ratio	0.1057 ± 0.0062 vs. 0.0887 ± 0.0041	*t*-test	0.056
Striatum	NE_DA_ratio	0.0461 ± 0.0158 vs. 0.0428 ± 0.0047	*t*-test	0.847
Striatum	3-MT	1.1767 ± 0.1606 vs. 1.2677 ± 0.1861	*t*-test	0.721
Striatum	3-MT_DA_ratio	0.0367 ± 0.0046 vs. 0.0277 ± 0.0026	*t*-test	0.135
Brainstem	NE	2.9158 ± 0.2772 vs. 2.8103 ± 0.3528	*t*-test	0.817
Brainstem	DOPAC	0.9783 ± 0.1084 vs. 1.3024 ± 0.0863	*t*-test	**0.0349**
Brainstem	HIAA	14.5029 ± 0.8499 vs. 15.1052 ± 0.8208	*t*-test	0.618
Brainstem	HVA	0.6920 [0.5892–0.9747] vs. 0.7722 [0.6104–1.0470]	Wilcoxon	0.743
Brainstem	5-HT	14.6312 ± 0.4686 vs. 15.1416 ± 0.5643	*t*-test	0.497
Brainstem	DA	1.4474 ± 0.0747 vs. 1.8236 ± 0.0654	*t*-test	**0.00265**
Brainstem	HIAA_5-HT_ratio	0.9730 [0.9337–1.0661] vs. 0.9927 [0.8900–1.0239]	Wilcoxon	0.963
Brainstem	HVA_DA_ratio	0.5132 ± 0.0569 vs. 0.4706 ± 0.0515	*t*-test	0.589
Brainstem	DOPAC_DA_ratio	0.7447 [0.6813–0.8359] vs. 0.6773 [0.6649–0.7189]	Wilcoxon	0.383
Brainstem	NE_DA_ratio	1.9907 ± 0.2490 vs. 1.3764 ± 0.1619	*t*-test	0.0647
Thalamus	NE	2.6171 ± 0.2916 vs. 2.8261 ± 0.4574	*t*-test	0.711
Thalamus	DOPAC	1.0061 ± 0.1881 vs. 0.9061 ± 0.0886	*t*-test	0.643
Thalamus	HIAA	14.1341 ± 0.6488 vs. 15.2321 ± 0.5604	*t*-test	0.227
Thalamus	HVA	1.2588 ± 0.0937 vs. 1.3182 ± 0.0703	*t*-test	0.622
Thalamus	5-HT	7.8124 ± 0.4626 vs. 7.9951 ± 0.3679	*t*-test	0.763
Thalamus	DA	5.2250 [4.3440–10.2294] vs. 4.8616 [3.3281–6.4735]	Wilcoxon	0.445
Thalamus	3-MT	0.7181 ± 0.1786 vs. 0.6387 ± 0.1374	*t*-test	0.741
Thalamus	HIAA_5-HT_ratio	1.8217 ± 0.0549 vs. 1.9181 ± 0.0833	*t*-test	0.36
Thalamus	HVA_DA_ratio	0.1939 ± 0.0469 vs. 0.3211 ± 0.0616	*t*-test	0.133
Thalamus	DOPAC_DA_ratio	0.1153 [0.1060–0.1392] vs. 0.2085 [0.1596–0.2639]	Wilcoxon	**0.00866**
Thalamus	3-MT_DA_ratio	0.0668 [0.0494–0.1342] vs. 0.1127 [0.1123–0.1131]	Wilcoxon	0.533
Thalamus	NE_DA_ratio	0.3747 ± 0.0819 vs. 0.5513 ± 0.0894	*t*-test	0.181
Cerebellum	NE	0.5689 [0.3719–0.6374] vs. 0.5002 [0.4150–1.9464]	Wilcoxon	0.755
Cerebellum	HIAA	2.0737 [1.6571–2.4096] vs. 2.0547 [1.9067–2.1380]	Wilcoxon	1
Cerebellum	HVA	0.2941 ± 0.1090 vs. 0.1842 ± 0.0155	*t*-test	0.42
Cerebellum	5-HT	1.1873 ± 0.1027 vs. 1.0281 ± 0.0760	*t*-test	0.237
Cerebellum	HIAA_5-HT_ratio	1.8565 ± 0.3382 vs. 1.9975 ± 0.1985	*t*-test	0.727

### Appendix A.2. Data Table for the Effect of 14-Day ICV Administration of 1.5 μL of 100 μM Marinobufagenin on the Activity of Monoaminoxidase B (MAO-B), Superoxide Dismutase (SOD), Catalase and Amount of Malonic Dialdehyde (MDA) in the Striatum and Prefrontal Cortex (PFC) of Animals

**Table A2:** The effect of 14-day ICV administration of 1.5 μL of 100 μM marinobufagenin on the activity of monoaminoxidase B (MAO-B), superoxide dismutase (SOD), catalase and amount of malonic dialdehyde (MDA) in the striatum and prefrontal cortex (PFC) of animals. Data are presented as Mean ± SEM, significant differences highlighted in bold.

**Structure**	**Enzyme**	**Activity, nmol/mg Protein/h (aCSF|MBG)**	**Test Used**	***p***
Striatum	MAO-B	3.24 ± 0.52|4.65 ± 0.25	*t*-test	**0.036**
PFC	MAO-B	3.89 ± 0.35|3.38 ± 0.41	*t*-test	0.368
**Structure**	**Enzyme**	**Activity, units/min/mg Protein (aCSF|MBG)**	**Test**	***p***
Striatum	SOD	0.06 ± 0.004|0.07 ± 0.004	*t*-test	0.180
PFC	SOD	0.07 ± 0.01|0.06 ± 0.004	*t*-test	0.131
**Structure**	**Enzyme**	**Activity, µmol/mg Protein/min (aCSF|MBG)**	**Test**	***p***
Striatum	catalase	29.97 ± 3.66|30.79 ± 3.85	*t*-test	0.879
PFC	catalase	25.40 ± 1.67|19.37 ± 3.69	*t*-test	0.18
**Structure**	**Substance**	**Amount, nmol/mg Protein (aCSF|MBG)**	**Test**	***p***
Striatum	MDA	84.05 ± 5.25|82.51 ± 6.39	*t*-test	0.856
PFC	MDA	91.70 ± 5.05|74.62 ± 3.08	*t*-test	**0.013**
